# Disparities in Racial and Ethnic Representation in Clinical Trials for FDA‐Approved Treatments of Endometriosis

**DOI:** 10.1111/1471-0528.18305

**Published:** 2025-07-23

**Authors:** Raanan Meyer, Jamila Piri, Neta Oranim, Raine Ngo, Gabriel Levin

**Affiliations:** ^1^ Division of Minimally Invasive Gynecologic Surgery, Department of Obstetrics and Gynecology Cedars Sinai Medical Center Los Angeles California USA; ^2^ The Dr. Pinchas Bornstein Talpiot Medical Leadership Program, Sheba Medical Center Ramat‐Gan Israel; ^3^ University of California, san Diego California USA; ^4^ Arizona State University Tempe Arizona USA; ^5^ Division of Gynecologic Oncology Jewish General Hospital, McGill University Quebec Canada

**Keywords:** access, barriers, clinical trials, diversity, equity, inclusion

Endometriosis affects approximately 10% of reproductive‐aged women, causing significant morbidity, including pelvic pain and infertility [1]. Historically, race was identified as a risk factor for endometriosis, with endometriosis predominantly identified among White women [2]. Despite its reported prevalence, racial and ethnic disparities in clinical trials evaluating treatments remain underexplored. Identifying potential enrolment disparities is critical to ensure equitable representation and assess treatment efficacy across racial and ethnic groups. We aimed to analyse enrolment rates by race and ethnicity in clinical trials that led to Food and Drug Administration (FDA) approvals for the treatment of endometriosis symptoms.

We conducted a cross‐sectional study analysing clinical trials that resulted in FDA approvals for endometriosis treatments between 1980 and 2025. Trials conducted outside of North America were excluded. Enrolment fractions were calculated by dividing the number of trial participants stratified by racial or ethnic group by the reported endometriosis prevalence for each group. Endometriosis prevalence was determined based on three pivotal epidemiological studies in the population of the United States(Supporting Information). Crude odd ratios (OR) and 95% confidence intervals (CIs) were calculated from 2 × 2 contingency tables to compare the enrolment fractions of minority participants to White participants. We performed a multistep analysis as follows: (1) studies that included White versus Black participants and (2) studies that included White, Black and Asian or Pacific Islander participants. A two‐tailed test was employed. Data analysis was performed using MedCalc version 23.1.6 (MedCalc Software bv, Ostend, Belgium) and GraphPad Prism version 6.1 (GraphPad Software, Boston, MA). A *p* value of <0.05 was considered statistically significant. As data for this study are publicly available (Supporting Information), Institutional Review Board (IRB) approval was not sought.

Nine clinical trials led to FDA approvals for endometriosis (Table S1). Of these, four studies reported race, and none reported ethnicity. Three trials were conducted in North America and were included; one trial conducted outside of North America was excluded (Figure S1). The total number of included participants was 3211. All trials were phase III and evaluated medications, including gonadotropin‐releasing hormone agonists, antagonists and progestins. Two trials categorised race into White, Black and Other groups. In these studies, Black participants were overrepresented compared to White participants (OR 1.86, 95% CI 1.37–2.50, Figure 1, Tables S2 and S3). One study additionally included Asian or Pacific Islander participants. In that study, Black participants were again overrepresented compared to White participants (OR 1.76, 95% CI 1.14–2.71), whereas Asian or Pacific Islander participants were underrepresented (OR 0.13; 95% CI 0.03–0.54). Racial disparity in clinical trials is a matter of interest to patients, clinicians, pharmaceutical companies and other stakeholders, and recent data suggest that clinical trials leading to FDA approvals do not adequately reflect the United States population [3, 4]. This study reveals inconsistent racial and ethnic reporting in clinical trials leading to FDA approval for endometriosis treatments. Among trials reporting race, Black participants were overrepresented, while Asian or Pacific Islander participants were underrepresented.

**FIGURE 1:**
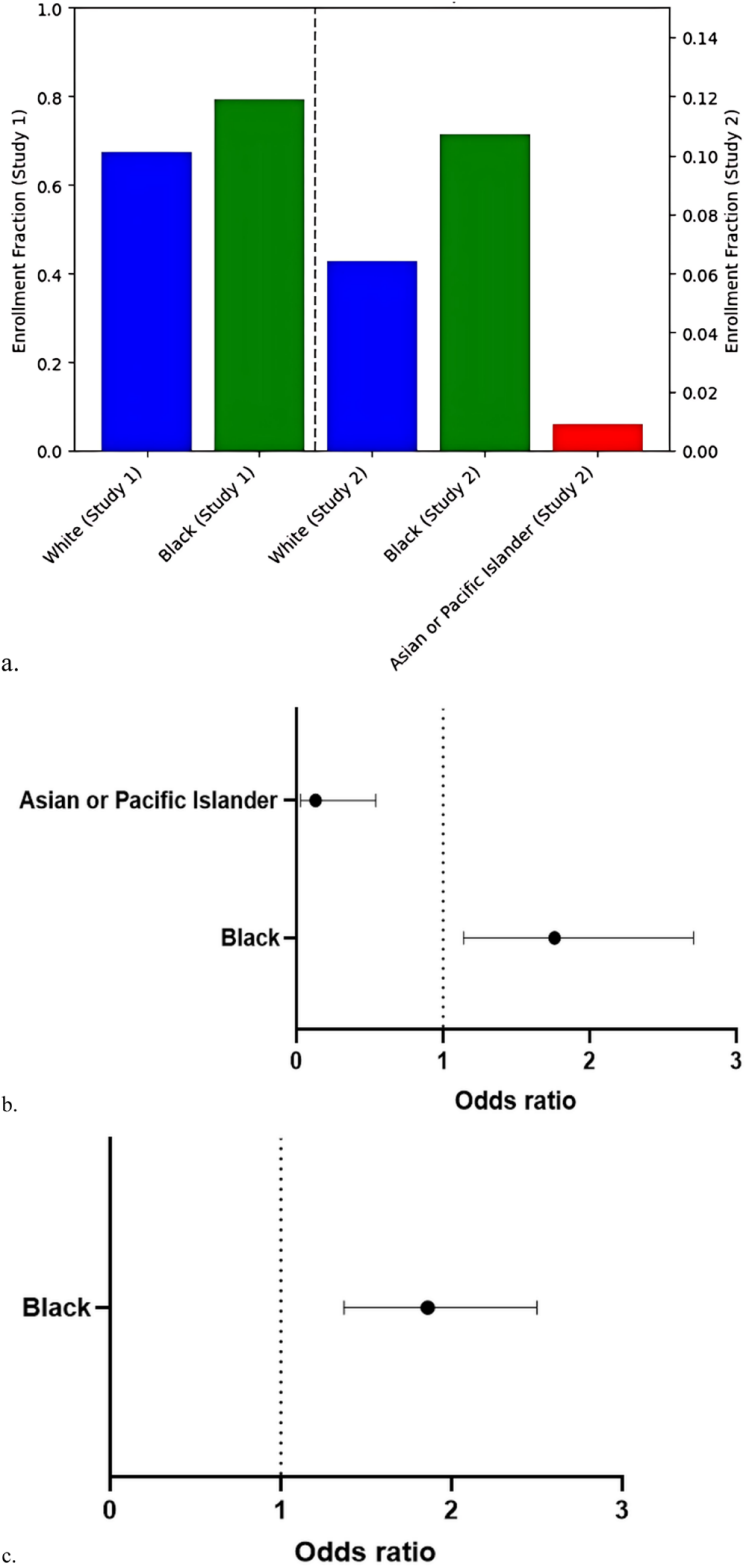
(a) Enrolment fractions by race. (b) Odds ratio of enrolment of Black compared to White race (reference). (c) Odds ratio of enrolment of Black and Asian or Pacific Islander compared to White race (reference).

Previous prevalence studies provide limited data on racial prevalence. However, a common finding is a significantly lower prevalence of Black women [5]. It is thus unexpected that Black individuals would be overrepresented in clinical trials for endometriosis treatment. The absence of ethnicity data further limits the understanding of disparities. The main limitations of this study include a lack of robust information on endometriosis prevalence by race, a small number of clinical trials included, and a focus on the North American population. Strengths include the focus on FDA‐approved treatments. Given the potential differences in disease presentation and treatment response across racial and ethnic groups, future trials should prioritise transparent and comprehensive racial and ethnic group reporting. Ensuring diverse representation is essential for generalising trial results and addressing potential inequities in endometriosis care.

## Author Contributions

R.M., G.L.: Conception, design, analysis and interpretation of data, drafting the article, critical revision of the article, final approval of the version to be published. J.P., N.O., R.N.: Acquisition of data, critical revision of the article, final approval of the version to be published.

## Disclosure

R.M., Intuitive Surgical; Claria, Medical consultant. All other authors have no disclosures.

*Source of the Study*: Cedars Sinai Medical Center.

## Conflicts of Interest

The authors declare no conflicts of interest.

## Supporting information


**Figure S1** Selection of study cohort.
**Table S1**. Clinical trials included and excluded.
**Table S2**. Characteristics of enrolled patients in the trials.
**Table S3**. Studies describing race proportion among endometriosis patients in the United States.

## Data Availability

The data that support the findings of this study are available from the corresponding author upon reasonable request.
