# Supplementary material for: Disparities in Racial and Ethnic Representation in Clinical Trials for FDA‐Approved Treatments of Endometriosis
Source: BJOG. 2025 Jul 23;132(13):2302–3. doi: 10.1111/1471-0528.18305 (PMC12592750; doi:10.1111/1471-0528.18305)
Supplement: Supplementary file 1 — Figure S1 Selection of study cohort. Table S1. Clinical trials included and excluded. Table S2. Characteristics of enrolled patients in the trials. Table S3. Studies describing race proportion among endometriosis patients in the United States. [file BJO-132-2302-s001.docx]

**Figure S1.** Selection of study cohort

**Table S1.** Clinical Trials included and excluded

| **Author and Year** | **Name** | **Phase** | **Agent** | **Setting** | **n** | **Location (participating country count in continent)** | **Race/Ethnicity** |
| --- | --- | --- | --- | --- | --- | --- | --- |
| **Included** |  |  |  |  |  |  |  |
| Schlaff et al, 2004[1] | Study 268 | III | Depot medroxyprogesterone acetate, Leuprolide | Endometriosis | 274 | North America (2) | Race |
| Taylor et al, 2018[2] | ELARIS EM-I, II | III | Elagolix | Endometriosis | 1686 | Africa (1), Australia (2), Europe (7), North America (2), South America (2) | Race |
| Giudice et al, 2022[3] | SPIRIT 1, 2 | III | Relugolix, Estradiol, Norethisterone | Endometriosis | 1251 | Africa (1), Australia (2), Europe (12), North America (2), South America (3) | Race |
| **Excluded** |  |  |  |  |  |  |  |
| Surrey et al, 1992 |  | III | Leuprolide, Norethindrone | Endometriosis | 20 | North America (1) | - |
| Dlugi et al, 1990 |  | III | Leuprolide | Endometriosis | 52 | North America (1) | - |
| Henzl et al, 1991 |  | III | Nafarelin | Endometriosis | 213 | North America (1) | - |
| Shaw et al, 1993 |  | III | Goserelin, Danazol | Endometriosis | 307 | Europe (8) | - |
| Buttram et al, 1996 |  | II | Danazol | Endometriosis | 220 | North America (1) | - |
| Crosignani et al, 2005 |  | III | Depot medroxyprogesterone acetate, Leuprolide | Endometriosis | 299 | Europe, Asia, Latin America and New Zealand* | Race |

* Information on the number of study locations was not available.

**Table S2.** Characteristics of enrolled patients in the trials

| **Race** | **Number enrolled** | **Disease prevalence** | **Enrollment Fraction** | **Odds Ratio** | **95% Confidence Interval** | **p Value** |
| --- | --- | --- | --- | --- | --- | --- |
| **Two studies categorizing White and Black** | | | | | | |
| **White** | 2624 | 3890 | 0.67% | Reference |  |  |
| **Black** | 219 | 276 | 0.79% | 1.86 | 1.37-2.50 | <.001 |
| **One study categorizing White, Black** **and Asian or Pacific Islander** | | | | | | |
| **White** | 237 | 3698 | 0.06% | Reference |  |  |
| **Black** | 25 | 233 | 0.11% | 1.76 | 1.14-2.71 | .011 |
| **Asian or Pacific Islander** | 2 | 223 | 0.01% | 0.13 | 0.03-0.54 | .005 |

**Table S3.** Studies describing race proportion among endometriosis patients in the United States

| **Study** | **Year** | **White** | **Black** | **Asian or Pacific Islander** |
| --- | --- | --- | --- | --- |
| Fuldeore et al [4] | 2017 | 2,394 | 148 | 58 |
| Christ et al [5] | 2021 | 1,304 | 85 | 165 |
| GiglioAyers et al [6] | 2024 | 192 | 43 | - |

**References**

1. Schlaff, W.D., et al., *Subcutaneous injection of depot medroxyprogesterone acetate compared with leuprolide acetate in the treatment of endometriosis-associated pain.* Fertil Steril, 2006. **85**(2): p. 314-25.

2. Taylor, H.S., et al., *Treatment of Endometriosis-Associated Pain with Elagolix, an Oral GnRH Antagonist.* N Engl J Med, 2017. **377**(1): p. 28-40.

3. Giudice, L.C., et al., *Once daily oral relugolix combination therapy versus placebo in patients with endometriosis-associated pain: two replicate phase 3, randomised, double-blind, studies (SPIRIT 1 and 2).* Lancet, 2022. **399**(10343): p. 2267-2279.

4. Fuldeore, M.J. and A.M. Soliman, *Prevalence and Symptomatic Burden of Diagnosed Endometriosis in the United States: National Estimates from a Cross-Sectional Survey of 59,411 Women.* Gynecol Obstet Invest, 2017. **82**(5): p. 453-461.

5. Christ, J.P., et al., *Incidence, prevalence, and trends in endometriosis diagnosis: a United States population-based study from 2006 to 2015.* Am J Obstet Gynecol, 2021. **225**(5): p. 500.e1-500.e9.

6. GiglioAyers, P., et al., *Demographic Correlates of Endometriosis Diagnosis Among United States Women Aged 15-50.* J Minim Invasive Gynecol, 2024. **31**(7): p. 607-612.
